# Longitudinal Motor-Developmental Outcomes in Infants with a Critical Congenital Heart Defect

**DOI:** 10.3390/children9040570

**Published:** 2022-04-16

**Authors:** Maaike C. A. Sprong, Marco van Brussel, Linda S. de Vries, Janjaap van der Net, Joppe Nijman, Johannes M. P. J. Breur, Martijn G. Slieker

**Affiliations:** 1 Center of Child Development, Exercise and Physical Literacy, Wilhelmina Children’s Hospital, University Medical Center Utrecht, 3508 AB Utrecht, The Netherlands; m.vanbrussel@umcutrecht.nl (M.v.B.); j.vandernet@umcutrecht.nl (J.v.d.N.); 2 Department of Neonatology, Wilhelmina Children’s Hospital, University Medical Center Utrecht, 3508 AB Utrecht, The Netherlands; l.s.devries-23@umcutrecht.nl; 3 Pediatric Intensive Care Unit, Wilhelmina Children’s Hospital, University Medical Center Utrecht, 3508 AB Utrecht, The Netherlands; j.nijman@umcutrecht.nl; 4 Department of Pediatric Cardiology, Wilhelmina Children’s Hospital, University Medical Center Utrecht, 3508 AB Utrecht, The Netherlands; h.breur@umcutrecht.nl (J.M.P.J.B.); m.g.slieker@umcutrecht.nl (M.G.S.)

**Keywords:** critical congenital heart disease, infants, children, cardiac surgery, neurodevelopmental outcomes, motor development, gross motor delay, Bayley-III

## Abstract

Infants with critical congenital heart defects (CCHDs) are at increased risk for neurodevelopmental delays. The early identification of motor delays is clinically relevant to prevent or reduce long-term consequences. The current study aims to describe the motor-developmental pathways of infants with a CCHD. Motor development was assessed in 215 infants and toddlers using the Dutch version of the Bayley-III. At 3 months (*n* = 165), 9 months (*n* = 188), and 18 months (*n* = 171) the motor composite scores were 97, 98, and 104, respectively. A motor composite score of ≤−2 SD was only seen in 2.4%, 0%, and 2.3%, respectively, with gross motor deficits being observed more often than fine motor deficits (12% vs. 0% at 18 months). Over 90% of infants who scored average at 9 months still did so at 18 months. The majority of infants with below-average gross motor scores (≤−1) at 9 months still had a below-average or delayed motor score (≤−2 SD) at 18 months. Abnormal gross motor scores (≤−2 SD) increased with age. Infants with single-ventricle physiology performed significantly (*p* ≤ 0.05) worse on both fine and gross motor skills at 9 and 18 months compared to infants with other CCHDs.

## 1. Introduction

Congenital heart disease (CHD) occurs in approximately 9 per 1,000 live births and corresponds to 1.35 million newborns worldwide every year [1]. CHDs are the leading cause of infant death from birth defects [2]. Around one-fourth of all CHDs require early diagnosis and surgical correction in the first months of life, e.g., hypoplastic left-heart syndrome, transposition of the great arteries, and tetralogy of Fallot, and are therefore considered critical [3]. The mortality rate of infants with a critical congenital heart disease (CCHD) has markedly declined over recent decades due to major advances in cardiac surgery and perioperative care [4].

Nowadays, approximately up to 93% of these infants with CCHD survive into adulthood [5]. Consequently, emphasis on research and clinical practice has shifted from acute morbidity and mortality to long-term neurodevelopmental outcomes. Neurodevelopmental disabilities are common in survivors of neonatal open-heart surgery, varying from mild and barely detectable to severe motor-developmental delays [6,7]. The causal pathways of such abnormal development appear to be multifactorial and are not fully understood. Therefore, systematic health observations throughout childhood are strongly recommended [8,9].

Numerous studies that investigated the development of infants with a CCHD applied a retrospective or cross-sectional study design and were part of studies including multiple congenital heart defects, varying from mild to severe, including infants with genetic anomalies and preterm-born infants [10]. Even though results differ, infants with CCHD are likely to be at higher risk for neurodevelopmental delays compared to healthy peers. Infants with a single-ventricle physiology (SVP) seem to be at the highest risk, especially those with hypoplastic left-heart syndrome (HLHS) [11,12]. These neurodevelopmental delays are of concern in relation to the longitudinal outcomes of these patients as they are at potential risk for future limited physical and educational achievements, reduced social interaction, and diminished quality of life [13,14,15].

Motor skills are essential aspects of overall development. Motor skills allow infants and children to explore their environment, enable them to master play skills and daily life skills (e.g., eating), and demonstrate independence through mobility. In addition, the ability to move and explore the environment affects perceptual, cognitive, and social learning. Early interventions, like active training of specific motor skills or offering activation strategies to parents, can positively influence the postoperative recovery and motor development of infants with a CCHD [16,17]. Therefore, early identification, differentiation, and prediction of motor outcomes are clinically relevant to intervene at an early stage so complications later in life can be averted or reduced.

Although there are several cross-sectional studies on motor development in children with a CHD, longitudinal (prospective) studies on motor development until (pre) school age and beyond in children with a CCHD are scarce [12,18]. Due to the natural variation of motor development in infants, serial motor assessment is necessary to accurately identify motor delay [19]. Motor skills are often assessed as a single entity and only a few studies report the difference between fine motor (FM) and gross motor (GM) skills [12]. Assessing both skills separately is relevant as GM delay can complicate participation in exercise and sports and result in a sedentary lifestyle and social isolation from peers. FM delay can lead to educational problems, especially in combination with cognitive impairments. Furthermore, the specification of the type of motor delay is important for parental counseling and to offer adequate interventions at an early stage. Therefore, the current study aims to longitudinally define total motor, GM, and FM development in term-born infants with a CCHD, without genetic anomalies, during the first 18 months of life using serial motor assessment and to analyze differences in motor performance between the different types of CCHDs. The outcomes of the current study could contribute to the knowledge of the longitudinal development of patients with a CCHD and provide more detailed information about infants at risk for motor-developmental delays.

## 2. Materials and Methods

### 2.1. Study Design

This study is part of a prospective, observational cohort study. Parents of infants with a CCHD who underwent cardiac surgery with cardiopulmonary bypass within the first 6 months of life between July 2011 and December 2019 at the Wilhelmina Children’s Hospital Utrecht, University Medical center Utrecht, The Netherlands, were invited to participate in developmental follow-up examinations from birth into adulthood as part of our standard clinical care. Parents were invited after the first cardiac surgery (with cardiopulmonary bypass) of their infant. Between 2011 and 2016, only infants with transposition of great arteries (TGA), tetralogy of Fallot (TOF), and single-ventricle physiology (SVP) were invited to participate; from 2016 onwards, infants with other types of CCHDs who underwent cardiac surgery with cardiopulmonary bypass within the first 6 months of life were also invited for follow-up.

### 2.2. Participants

**Figure 1:**
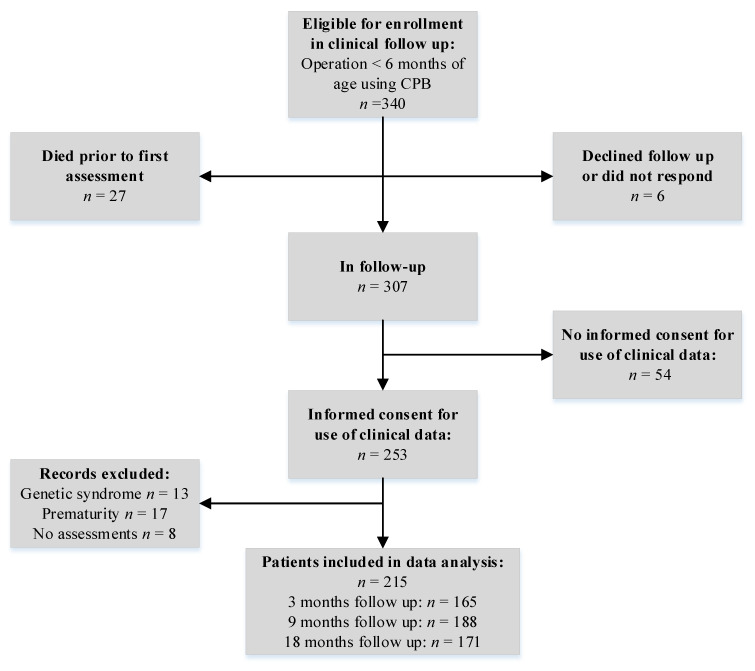
Enrollment flow chart.

During the study period, a total of 340 infants with a CCHD were eligible for enrollment in our developmental outpatient clinic. Of all eligible patients, 307/340 (90%) enrolled in the clinical follow-up program (Figure 1). The exclusion criteria from the current data analysis included confirmed genetic anomalies including trisomy 21, 22q11 deletion or CHARGE syndrome, and prematurity, which is defined as birth before 36 weeks of gestation, because these infants are at increased risk of developmental disorders regardless of their CHD [20,21,22,23]. Infants with acquired motor deficits unrelated to CHD (e.g., sequelae of meningitis, nerve or muscle trauma) were also excluded from data analysis. Data on patient characteristics and clinical variables were obtained prospectively. A pediatric cardiologist (M.G.S.) reviewed the medical records to confirm the cardiac diagnoses.

### 2.3. Outcome Assessment

At 3, 9, and 18 months, all infants underwent neurodevelopmental examination by an experienced developmental pediatrician/neonatologist and a pediatric physical therapist (M.C.A.S.) with >10 years of experience in developmental assessment. Assessments took place at least 6 weeks after cardiac surgery. When patients were still clinically admitted at that time, their clinical condition was sufficient to temporarily leave the ward in order to visit the outpatient clinic. The physical examination included a standard neurological examination according to the recommendations from the Cardiac Neurodevelopmental Outcome Collaborative [9]. The examination was performed at each follow-up visit, with particular attention to muscle tone (overall, axial, limbs), symmetry (signs of asymmetry), and motor skills [9]. In addition, reflexes, cranial nerves (eye contact/tracking movements, facial motor function), and functional muscle strength (arms/legs) were investigated.

The Dutch version of the Bayley Scales of Infant Development, Third Edition, (Bayley-III-NL) was used for evaluation of motor development at 3, 9 and 18 months [24]. The Bayley-III-NL is a standardized assessment tool to assess the development of children between 1 and 42 months of age in five different domains: cognitive, language, motor, social–emotional, and behavior. In this study, only the motor domain was analyzed. In contrast to other motor-assessment tools [25,26], the Bayley-III motor scale consists of a separate score for fine motor skills (FM scaled score) and gross motor skills (GM scaled score). Both scaled scores are combined in the motor composite score. The Bayley-III-NL is a practical assessment tool to identify infants and toddlers with an abnormal motor development. This tool allows the user to compare the gross and fine motor skills of infants and toddlers with normative data in order to discriminate between typical development and/or an abnormal development. In addition, the tool can also be used to evaluate motor development between 0 and 42 months. Therefore, the Bayley-III-NL was used to assess motor development at 3, 9 and 18 months. Motor composite scores (mean 100, SD 15) were used, and the separate FM scaled scores and GM scaled scores were analyzed (mean 10, SD 3). Furthermore, classifications based on composite and scaled scores were applied. A motor composite score ≤ 70 or a fine or gross motor scaled score ≤ 4 (2 SD below the mean) indicates an “abnormal motor score” or “(significant) motor delay”. Gross and FM scaled scores were categorized as “below average” when motor scores were between 2 SD and ≤ −1 SD and “(above) average” when motor scores were >−1 SD. The Bayley-III-NL classification system was used to classify motor development as “average” (motor composite score ≥ 90), “below average” (motor composite score ≤ 89), or “delayed” (motor composite score ≤ 79).

### 2.4. Statistical Analysis

Continuous variables are presented as mean ± SD for normal distributions. The normality of the distribution of continuous variables was tested with the Kolmogorov–Smirnov test and visualized by the skewness and kurtosis of histograms. If not normally distributed, medians with interquartile range (IQR) are reported. Categorical variables are presented as frequencies and percentages. Differences in parametric, non-parametric, and dichotomous outcomes were analyzed using ANOVA, Kruskal–Wallis, or Chi-square test, respectively. A *p*-value < 0.05 was considered significant. Statistical analyses were performed with IBM SPSS^®^ version 25.0 (SPSS, Chicago, IL, USA).

## 3. Results

As already indicated in the methods, of all the eligible patients, 307/340 (90%) enrolled in the clinical follow-up program (Figure 1). Twenty-seven out of 340 infants (8%) died prior to the first examination, and parents of six infants (2%) declined the follow-up, were unavailable, or did not respond. The parents of fifty-four infants (18%) of the 307 follow-up participants did not consent to the use of their clinical data for research purposes.

The group of infants who declined the follow-up or did not consent to the use of their clinical data differed from the included infants in terms of age at surgery (40 days vs. 9 days) (*p* < 0.001) and the diagnosis distribution (*p* = 0.006). There were fewer infants with TOF (31.7% vs. 18%), fewer infants with aortic arch pathology (20% vs. 9%) and significantly more infants with a TGA (18% vs. 43%) in the study population. The number of infants with SVP did not differ substantially (18% vs. 19%). There was no significant difference in sex distribution (67% vs. 63% boys) (*p* = 0.63) between the groups.

A total of 215 infants with a CCHD fulfilled the inclusion criteria (Figure 1), 165 of whom completed the motor assessments at 3 months, 188 at 9 months, and 171 at 18 months. In total, 130 participants (60%) completed all three assessments at 3, 9, and 18 months. A total of 46 (27%), 18 (9%) and 12 (7%), respectively, had no measurement at age 3, 9 or 18 months. The reasons for missing data were no-shows or because the clinical or behavioral state did not allow for a developmental assessment. At three months of age, some of the infants had not yet undergone their surgical correction and were therefore not yet enrolled in the follow-up (especially TOF patients), since the infants were invited to the developmental follow-up after their heart surgery had taken place. Patient characteristics, including CCHD diagnoses, are presented in Table 1.

### 3.1. Developmental Outcomes

At 3, 9, and 18 months, the mean motor composite scores, assessed with the Bayley-III-NL, were 96 (IQR 88–107), 98 (IQR 89–107), and 107 (IQR 98–112), respectively. A significant motor delay (total motor score < 70) was seen in 4 (2.4%), 0 and 4 (2.3%) infants, respectively.

In our population, seven infants (3.3%) developed signs of cerebral palsy (CP) such as abnormal muscle tone, strength, reflexes, and signs of asymmetry during the first eighteen months. Only one of these infants completed all three assessments. Six infants showed signs of unilateral paresis such as unilaterally reduced motor coordination, which is an asymmetrical muscle tone or asymmetrical use of lower and/or upper extremities, at one or more consecutive moments. One of the infants showed signs of mild spastic diplegia (mildly increased tone in both legs in combination with poor total motor coordination) at 18 months. The infants with signs of CP were included in the group analyses as the group results did not significantly change after excluding them from the analyses.

#### 3.1.1. Fine Motor Development

The mean Bayley-III-NL FM scaled scores were 9.4 (±2.9), 10.5 (±1.6), and 11.2 (±2.0) at 3, 9, and 18 months, respectively. Five infants (3%) had FM scaled scores at or below 4 (≤−2 SD) at 3 months. None of the infants had abnormal FM outcomes at 9 or 18 months.

#### 3.1.2. Gross Motor Development

**Figure 2:**
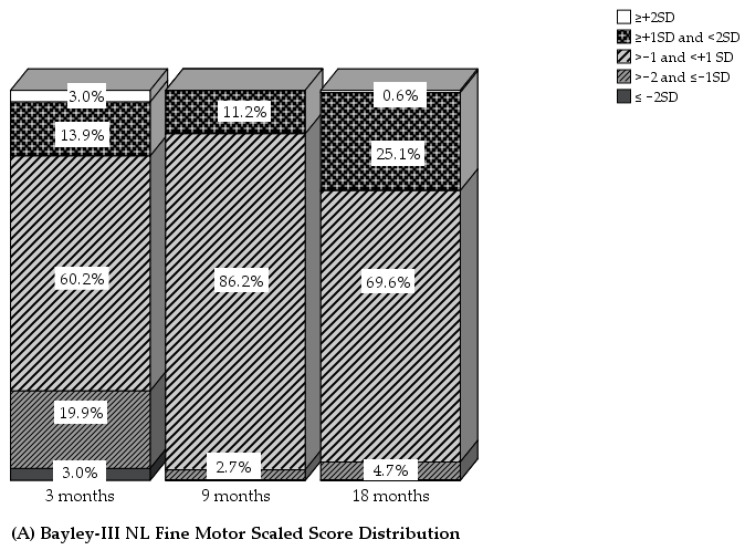

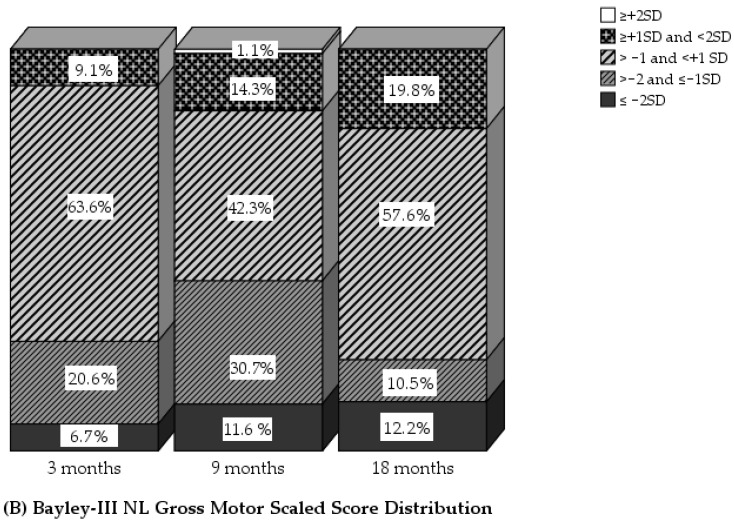
Bayley-III motor classifications of fine motor (**A**) and gross motor (**B**) development at 3, 9, and 18 months in children with a critical congenital heart defect (CCHD).

At 3, 9 and 18 months, the Bayley-III-NL mean GM scaled scores were 9.2 (±2.7), 8.7 (±3.2), and 9.7 (±3.3), respectively; 7%, 12%, and 12% of the infants had GM scaled scores at or below 4, which is 2 SD below the population mean at 3, 9, and 18 months, respectively (Table 2). Detailed information about the distribution of GM and FM classifications at 3, 9, and 18 months is displayed in Figure 2.

### 3.2. Longitudinal Motor Development 0–18 Months

One hundred and thirty infants with a CCHD completed all motor assessments at 3, 9, and 18 months. Seven of them (5%) showed a one-time total motor delay (<−2 SD) and four infants (3%) showed a delayed motor development at two consecutive moments between 0 and 18 months. In terms of GM delays, 16 out of 130 infants (12%) showed a one-time GM delay and 7 infants show a delayed GM development at two consecutive time-points between 0 and 18 months. None of the infants had a delayed total motor, FM, or GM development at all times.

#### 3.2.1. Total Motor Trajectories

Of the 89 infants whose motor skills were classified as “average” on the Bayley-III at 3 months, 81 (91%) infants still showed an average motor development at the age of 18 months.

The motor development of 28 (82%) of the 34 infants who scored “below average” at 3 months were normalized at 18 months. In 4 (12%), the motor classification remained the same, and 2 (6%) had a significant motor delay at 18 months. Of the 7 infants who had a “significant motor delay” at 3 months, only 1 (14%) still showed a significant motor delay at 18 months.

**Figure 3:**
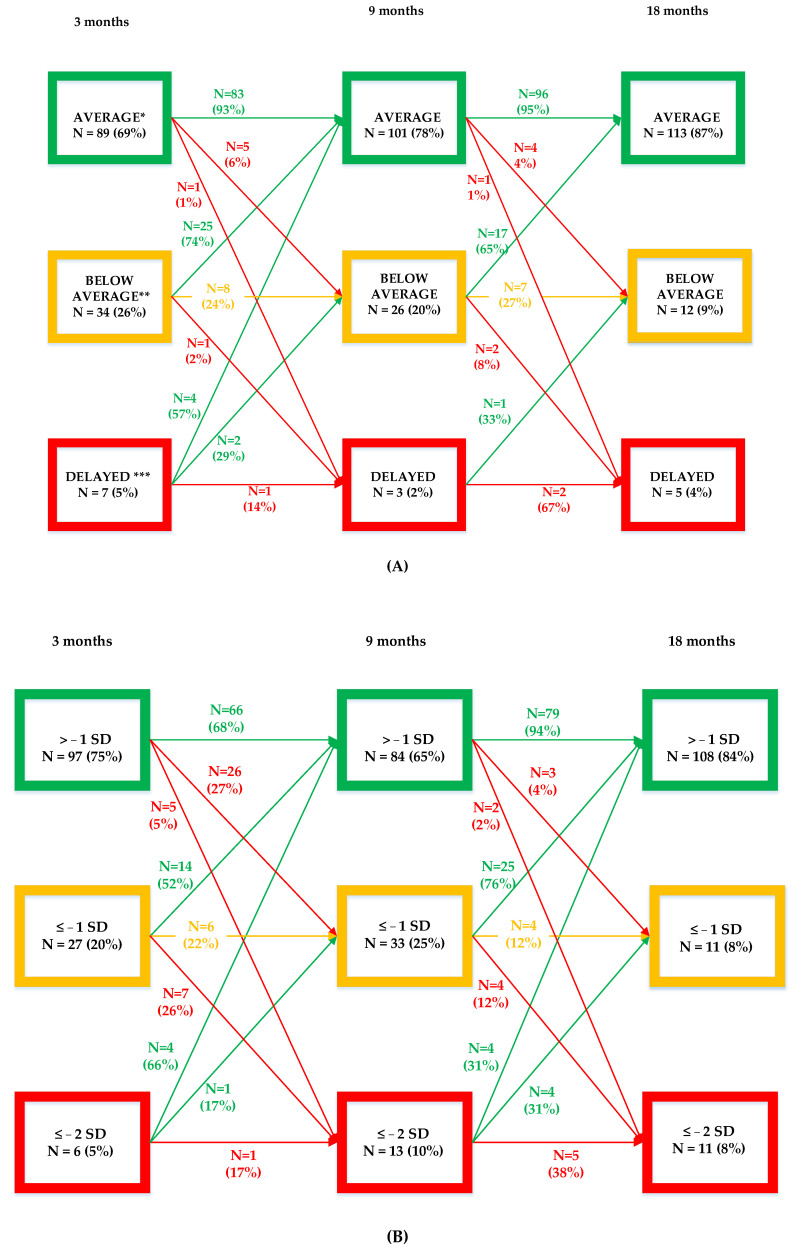

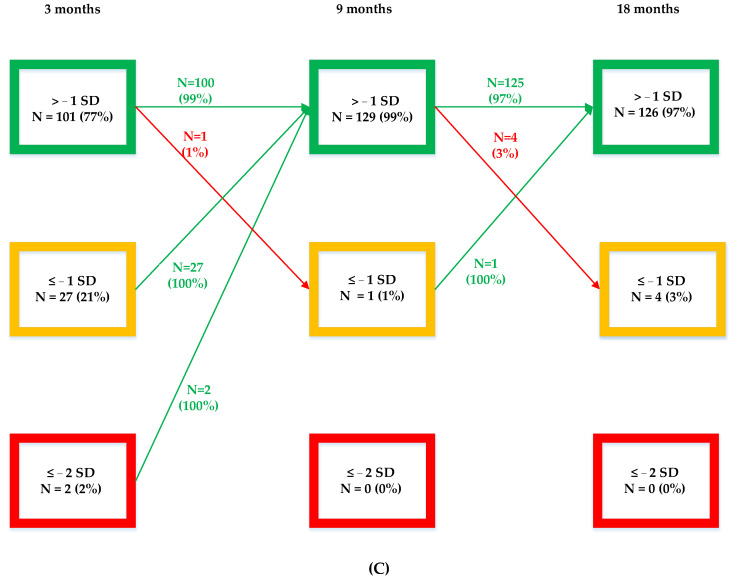
(**A**–**C**). Bayley-III development trajectories of total motor development, gross motor, and fine motor development at 3, 9, and 18 months. * Both total motor scores that fall within the classification “superior” (120–129*)* “high average” (110–119) or “average” (90–109) are displayed within the group “average”. ** Total motor scores between 80 and 89 are shown in the “below average” group. *** Both scores that fall within the classification “borderline” (70–79) and “extremely low” are displayed within the “delayed” group. Bayley-III = Bayley Scales of Infant and Toddler Development, Third Edition.

All infants who scored < −2 SD at 9 months still scored ≤ −1 SD or ≤ −2 SD at 18 months. For more detailed information about motor-developmental trajectories between 3 and 9, and 9 and 18 months, see Figure 3A.

#### 3.2.2. Gross Motor Trajectories

Of the 97 of the original 130 infants who had an average GM development at 3 months, 86 (89%) also had an average GM development at 18 months. Six of the initially average-scoring infants (6%) had a gross motor delay (≤−2 SD) at 18 months. In three out of the six infants (50%) who showed delayed GM skills at 3 months, GM development had normalized to “average” at 18 months. Only one (17%) of the six infants still had a GM delay. Thirty-eight percent of infants who showed a GM delay at 9 months still did so at 18 months. For more details about GM developmental trajectories between 3 and 9, and 9 and 18 months, see Figure 3B.

Of the 13 infants with a GM delay at 9 months, 9 infants already had physiotherapy, otherwise physiotherapy was started. In one third (*n* = 3) the GM skills fully normalized, and in one third (*n* = 3) the motor score improved and could be classified as “below average”. For the remaining three, (33%) a GM delay persisted in spite of the intervention. In the four infants where the natural course was awaited, two achieved an equal “delayed” score, one improved to “below average” and one to “average” at 18 months.

#### 3.2.3. Fine Motor Trajectories

Of the 101 infants who scored average on the Bayley-III-FM at 3 months, 99 (98%) still did so at 18 months. Two (2%) of them had below-average (−1 SD) FM development at 18 months.

Of the 29 infants who scored below average (*n* = 27) or even delayed (*n* = 2) at 3 months, only 2 (7%) still showed a below average FM development at 18 months. None of the infants had an FM delay at 18 months. For details about FM developmental trajectories, see Figure 3C.

### 3.3. Motor Development per Diagnosis Group

**Figure 4:**
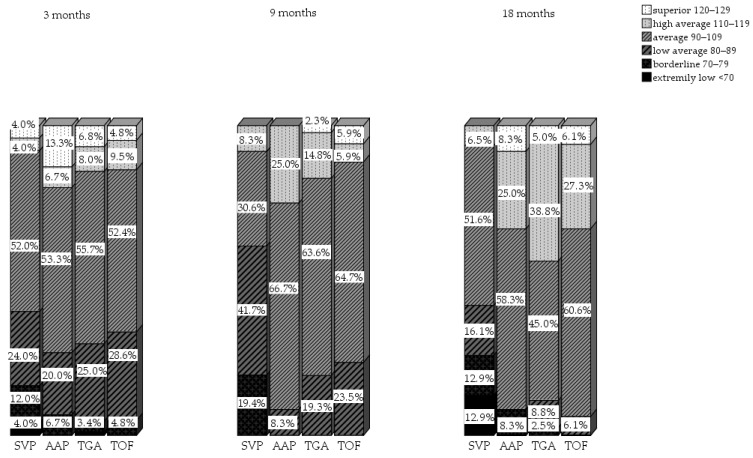
Bayley-III NL total motor development at 3, 9, 18 months specified per diagnosis group. SVP = single-ventricle physiology, TGA = transposition of great arteries, AAP = aortic arch pathology, TOF = tetralogy of Fallot.

When the results of the different diagnosis groups were analyzed separately, there was a significant difference in motor outcomes between infants with SVP and the other CCHD diagnosis groups at all ages (*p* < 0.05). The motor scores of infants with SVP were worse than the motor scores of infants in other diagnosis groups (Table 2). For most motor outcomes, children with HLHS did not differ significantly from children with other types of SVP. However, at the age of 3 months, the GM-scaled scores of infants with HLHS were significantly lower than those of infants with other types of SVP (GM-SS 7.08 vs. 10.23) (*p* = 0.03). Furthermore, at the age of 9 months, total motor scores were significantly lower in infants with HLHS compared to those with other types of SVP (86 vs. 95) (*p* = 0.047). Moreover, when the motor outcomes of children with a TGA were analyzed in more detail, the motor outcomes of infants with a TGA, who had undergone balloon atrioseptostomy (BAS), did not differ significantly from those of infants with a TGA who had not undergone BAS. Although the mean motor outcomes and distribution per motor classification for both total motor, FM and GM scaled scores were within the normal range, the number of infants with abnormal GM motor outcomes (≤−2 SD) was higher compared to the normal population in all groups at all ages. The percentages of infants scoring ≤ −2 SD increased as they aged. At the ages of 3, 9, and 18 months, 8%, 30.6%, and 32.2% of infants with SVP had a GM scaled score ≤ −2 SD, respectively. Detailed information per diagnosis group about the distribution of total motor classifications at 3, 9, and 18 months is displayed in Figure 4.

## 4. Discussion

This study aimed to longitudinally define total, gross, and fine motor development in an unselected prospective cohort of infants with a CCHD during the first 18 months of life using serial motor assessments and to explore differences in motor performance between the different types of CCHD.

The most remarkable result was the increasing number of infants with abnormal GM outcomes (≤−2 SD) at all consecutive moments. Within the abnormal GM outcomes, infants with SVP performed significantly worse than other CCHD types. The percentages of infants with SVP and an abnormal GM score increased with age, from 8% at 3 months up to 32.2% at 18 months. The majority of infants with CCHD and delayed total motor or GM development at 9 months still scored below average or delayed at 18 months. Nevertheless, in the overall study population, the mean motor outcomes for both total motor, FM and GM skills were within the normal range, and normally developing infants appeared stable over time: 90% of infants who achieved a mean motor composite score, FM scaled score, or GM scaled score at 9 months, still did so at 18 months.

Only a few other studies were found in which motor development was longitudinally analyzed [27,28,29]. Improvements in total motor abilities in the first years of life were found in all studies, which is in line with our findings. Mussato et al. [29] did find lower mean motor composite scores, which may be explained by the inclusion of preterm infants in their study.

The number of infants with abnormal GM skills (≤−2 SD) was higher compared to the normal population and increased in all diagnostic groups between 3 and 18 months. These lower GM scores are in agreement with previous studies that found poorer GM development compared to FM development in infants with CHD [30,31]. This indicates the importance of assessing and interpreting FM and GM developmental scales separately. To the best of our knowledge, the increasing number of infants with abnormal gross motor outcomes has not been previously described in the literature. Only a few comparable studies of a longitudinal character were found in which, in addition to an analysis of total motor outcomes, a distinction was made between fine and gross motor skills in infants between 0 and 18 months. Roberts et al. [28] showed, in contrast to our results, a decreasing number of infants with abnormal gross motor development between 6 and 12–18 months. The difference in measurement moments and the use of a different assessment tool, as gross motor development was measured with the Alberta Infant Motor Scale (AIMS) in this study, may explain the different results.

In our study, a relatively small number of infants with CCHD scored an abnormal (≤−2 SD) motor composite score (up to 2.4%) or FM scaled score (up to 3%). Normally, scores below these cutoffs occur in only 2.3% of the population [31]. The low prevalence of abnormal motor scores and mean motor scores in our population is in contrast to previous studies that described lower Bayley Scales of Infant Development-II (BSID-II) or Bayley-III total motor composite scores at one year, [18,32,33] and reported the prevalence of motor-developmental delay (≤−2 SD) of 10 to 20% [34,35]. An explanation for this discrepancy might be that our study population was monitored at the outpatient clinic as part of standard care and not by clinical indication. Following these consultations, parents were given advice on how to stimulate and facilitate the development of their infant and if needed, infants were referred to a pediatric physiotherapist. Infants growing up with a CCHD are often overprotected and are less challenged by parents, caregivers, and teachers as a result of lower perceived health [36,37]. The consistent advice to stimulate motor development might have contributed to a relatively low prevalence of motor delay in our population. Lastly, it is known that the Bayley-III might underestimate motor-developmental delay after early complex cardiac surgery in comparison with Bayley I & II [31,38]. Moreover, intercultural differences could explain the difference between our findings and results from other countries as standardized motor-development-assessment tools have limited validity in cultures other than that in which the normative sample was established. Their application can result in under- or over-referral for services [39,40]. Dutch normative data were applied to interpret the results of the Bayley-III. There are clinically relevant differences between the Dutch norms and the US norms, which may explain the difference in outcomes with studies from other countries.

Infants with SVP showed “average” mean total motor outcomes as well, which is in contrast to previous findings, where mean total motor scores ≤ −1 SD were reported [41,42]. However, infants with SVP showed significantly lower FM, GM, and total motor outcomes compared to infants with other cardiac diagnoses. At 18 months, over 30% of infants with SVP had a GM delay. Lower GM scores can be due to various factors, e.g., (higher) number of surgeries, (longer) hospital stays and long-term oxygen-saturation levels below 85% [35,43,44,45,46,47]. These factors increase the risk of white-matter injury during neonatal heart surgery, due to immaturity and smaller cortical volumes after birth [48,49]. For example, infants with a TGA generally need one surgery in the first year of life, as well as a BAS in more than half of these infants. On the other hand, infants with an SVP at the age of 18 months, in general already had at least two operations; the aorto-pulmonary shunt (AP-shunt) operation and the partial cavo-pulmonary connection (PCPC). In addition to these two operations, these children often undergo one or more cardiac catheterizations in the first years. The third operation, which is needed to complete the Fontan circulation (total cavo-pulmonary connection), usually takes place after the second year of life. Because the Fontan circulation is not yet completed in most infants by the age of 18 months, the majority of children with SVP still have oxygen saturation levels of around 80%, which can result in reduced energy and activity levels as well as the increased risk of white-matter injury.

Multiple authors describe that muscle hypo- or hypertonia, which correlates with abnormal MRI findings, partially explains the GM problems of infants with severe CHD [7,30]. As a brain MRI was only performed on clinical indication in this cohort, we were unable to relate brain injury to motor outcomes. Nevertheless, in our population, seven infants showed clinical signs of cerebral palsy between 0 and 18 months. The age of 18 months may be too early to diagnose infants with a milder form of CP, so the number may be a little higher. In contrast to infants with SVP, a significantly smaller number of infants with TGA, TOF, or AAP had GM scores ≤ −2 SD at the age of 18 months (6.3%, 6.1%, and 8.3%, respectively). These infants generally undergo only one operation in their neonatal period, resulting in less frequent and shorter hospitalizations, and the literature describes less frequent and less severe brain damage in these infants. However, the number of infants with TGA, TOF, or AAP scoring ≤ −2 SD on GM skills was still almost three times higher compared to the normal population.

The increasing number of infants with GM developmental delays between 3 and 18 months is concerning as motor development could have significant long-term implications for social inclusion and quality of life. In addition, motor development appears to be an independent predictor of exercise capacity, activity level, level of participation in sports, and the frequency of sports activities in childhood throughout adolescence [8,14,15,50,51]. A major threat to cardiovascular health in children with CHD is a global lack of physical activity and sedentary behavior. The effects of inactivity are profound in several domains (i.e., physical, emotional, intellectual, financial) [52]. In addition, cardiopulmonary exercise capacity is a strong predictor of mortality, morbidity, and hospitalization [53] in adults with CHD. Improvements in motor skills at a young age are feasible, which can result in increased physical activity and sports participation during childhood and adolescence. The improvement of (gross) motor development could therefore be an important target for early interventions to prevent or diminish motor- and exercise-related problems and to improve participation with peers, exercise capacity, and physical activity in order to protect their current and future cardiovascular health [54,55]. Although the understanding of the underlying mechanisms and possible risk factors has improved in recent years, the full interplay between these factors is still not clear, allowing little progress in the field of therapeutic options. Considering the high incidence of GM delays, it is important to identify the underlying causes and risk factors. The need for strategies to improve modifiable risk factors and early therapeutic options to prevent or reduce GM delays is high and should also be the focus of future research, especially in infants with SVP. Additional research to identify risk factors is in progress and will be published separately. Furthermore, the gross and fine motor development of children with a CCHD should be closely monitored and in a structured manner from birth until adolescence or even young adulthood. Because of the increasing number of infants with abnormal gross motor scores, we recommend these infants should be referred for pediatric physiotherapeutic interventions to support their (gross) motor development in an accessible way.

Large prospective studies in unselected populations of infants with a CCHD are scarce. To the best of our knowledge, this is one of few single-center studies in which such a specific group of term-born infants without underlying genetic anomalies was followed longitudinally, which can be seen as a strength of our study. Based on the results concerning longitudinal motor trajectories of infants with a CCHD, a cautious prognosis can be made about the expected development at 18 months and thus enables a more targeted recommendation of early intervention at a young age. Another strength of our study is that, in addition to population means, the prevalence of delays of total motor development, GM, and FM skills at the group level and for specific types of CCHD was described, giving us more information about which motor skills are most affected.

Although it is well known that children with CCHD show delays in various developmental domains, [7] this article focused on the motor development of infants between 3 months and 18 months. As a result, we do not yet have information about whether we are looking at an isolated motor delay or an overall developmental delay. However, the content of our follow-up is in line with the guidelines of Ware et al. [9], which recommend investigating motor development from the age of six months onward and cognitive and language development from the age of 18 months. In our center, these assessments are performed at the age of 24 months and are therefore beyond the scope of this dataset. Another limitation might be that only diagnosis groups of which more than 10 infants were represented per group were included in the analyses. As a result, the developmental course of infants with other, less common congenital abnormalities such as truncus arteriosus and TAPVC remains unclear. In addition, missing data at the age of 3 months were not random, which may have had an impact on the results in both a positive and a negative way; data at 3 months were missing because some infants had a complicated clinical condition that did not allow for a developmental assessment. Other infants, on the other hand, had a less severe heart defect (e.g., TOF) and underwent surgery after the age of 3 months; they visited the outpatient clinic for the first time at 9 months.

## 5. Conclusions

This study highlights that the mean motor development of term-born infants with a CCHD without an underlying genetic anomaly is within the normal range and relatively stable in the first 18 months of life. Almost all infants who scored within the normal range at 9 months still do so at 18 months, and the majority of infants with delayed motor development at 9 months also persist to score below average or delayed at 18 months. Furthermore, the number of infants with abnormal gross motor outcomes (≤−2 SD) increases over time in all diagnosis groups, particularly in those with SVP. These latter findings stress the importance of a longitudinal evaluation of motor skills in infants with CCHD, especially in those with an SVP or low motor outcomes at the 9-month assessment. Because early motor development appears to be an independent predictor of exercise capacity, physical activity, participation in daily living, and the frequency of sports activities in childhood, improving motor skills in infants who demonstrate low motor outcomes at a young age should be an important target for early interventions in order to protect their current and future cardiovascular health.

## Figures and Tables

**Table 1:** Patient characteristics.

Patient Characteristics and Clinical Data	Total (*n* = 215)
Male	136 (63)
Birth weight, gram	3422 ± 564
Gestational age, weeks	39.4 ± 1.34
Apgar score 5 min	8.8 ± 1.2
Prenatal diagnosis	147 (69)
Balloon Atrioseptostomy (BAS)	59 (27)
Age at surgery (days)	9 (7–21)
Total intubation duration pre/per/post-surgery (hours)	99 (47–195)
Aortic Cross Clamp (ACC) time (minutes)	79 (62–97)
Cardio Pulmonary Bypass (CPB) time (minutes)	136 (108–167)
Low-Cardiac-Output Syndrome (LCOS) pre-surgery	25 (12)
Low-Cardiac-Output Syndrome (LCOS) post-surgery	44 (21)
Length of hospital stay (days)	19 (14–28)
Intensive-Care-Unit stay (days)	9 (5–14)
Cardiac Diagnosis	
Transposition of Great Arteries (TGA)	94 (44)
Congenital Corrected TGA (ccTGA)	1 (1)
Tetralogy of Fallot (TOF)	39 (18)
Single-Ventricle Physiology (SVP)	40 (19)
Truncus Arteriosus	3 (1)
Hypoplastic Left-Heart Complex	9 (4)
Aortic arch pathology	20 (9)
Total Anomalous Pulmonary Venous Connection (TAPVC)	5 (2)
Others	4 (2)
Age at follow up 3 (months*)*	3.3 (3.0–3.6)
Age at follow up 9 (months*)*	9.6 (9.0–10.4)
Age at follow up 18 (months*)*	18.9 (18.1–19.6)

Data are presented as mean +/− standard deviation (normally distributed) or as median with 25th/75th centiles (not normally distributed) or as a number with a percentage.

**Table 2:** Motor outcomes of infants and children with a CCHD at 3, 9, and 18 months: A comparison between single-ventricle physiology (SVP) and other diagnosis groups.

	Total	SVP	TGA	TOF	AAP	*p*-Value
**3 months**	*n* = 165	*n* = 25	*n* = 88	*n* = 21	*n* = 15	
**Bayley-III**						
Motor composite score	96.6 (13.3)	93.8 (13.0)	97.9 (12.4)	96.8 (13.5)	100.8 (14.9)	0.055
Fine motor scaled score	9.4 (2.9)	8.8 (2.7)	9.5 (2.8)	10.1 (3.3)	10 (2.8)	0.130
Gross motor scaled score	9.2 (2.7)	8.7 (2.8)	9.6 (2.6)	8.7 (2.2)	9.9 (3.1)	0.063
PDI ≤ −2 SD	4 (2.4)	1 (4)	1 (1.1)	0 (0)	0 (0)	
FM SS ≤ −2 SD	5 (3)	1 (4)	1 (1.1)	1 (4.5)	0 (0)	
GM SS ≤ −2 SD	11 (6.7)	2 (8)	5 (5.7)	1 (4.8)	1 (6.7)	
Discrepancy GM < FM	28 (17)	4 (16)	14 (15.9)	6 (28,6)	3 (20)	
**9 months**	*n* = 188	*n* = 36	*n* = 88	*n* = 34	*n* = 12	
**Bayley-III**						
Motor composite score *	98.3 (11.6)	91.2 (12.3)	100.6 (10.4)	99.5 (11.1)	102.1 (9.7)	0.001 *
Fine motor scaled score *	10.47 (1.6)	9.9 (1.6)	10.5 (1.4)	10.9 (1.7)	11.6 (1.6)	0.046 *
Gross motor scaled score *	8.7 (3.2)	6.8 (3.5)	9.4 (3.0)	8.6 (2.9)	8.8 (2.8)	0.001 *
PDI ≤ −2 SD	0 (0)	0 (0)	0 (0)	0 (0)	0 (0)	
FM SS ≤ −2 SD	0 (0)	0 (0)	0 (0)	0 (0)	0 (0)	
GM SS ≤ −2 SD	22 (11.7)	11 (30.6)	5 (5.7)	2 (5.9)	1 (8.3)	
Discrepancy GM < FM	57 (30.3)	19 (52.8)	19 (21.6)	9 (26.5)	6 (50)	
**18 months**	*n* = 171	*n* = 31	*n* = 80	*n* = 33	*n* = 12	
**Bayley-III**						
Motor composite score *	103.6 (12.7)	93.2 (14.9)	106.61 (11.1)	105.9 (9.7)	105.4 (11.3)	<0.000 *
Fine motor scaled score *	11.20 (2.0)	9.9 (2.5)	11.44 (1.6)	11.6 (1.7)	11.9 (2.4)	0.002 *
Gross motor scaled score *	9.7 (3.3)	7.45 (3.7)	10.63 (3.1)	10.2 (2.8)	9.6 (2.6)	0.001 *
PDI ≤ −2 SD	4 (2.3)	4 (12.9)	0 (0)	0 (0)	0 (0)	
FM SS ≤ −2 SD	0 (0)	0 (0)	0 (0)	0 (0)	0 (0)	
GM SS ≤ −2 SD	21 (12.2)	10 (32.3)	5 (6.3)	2 (6.1)	1 (8.3)	
Discrepancy GM < FM	43 (25.3)	12 (38.7)	15 (18.8)	8 (25)	3 (25)	

Data are presented as mean (+/− standard deviation) or as a number with a percentage. * Statistically significant differences between diagnosis groups, determined by ANOVA, *p*-value < 0.05. CCHD = critical congenital heart disease, SVP = single-ventricle physiology, TGA = transposition of great arteries (TGA), AAP = aortic arch pathology, TOF = tetralogy of Fallot, Bayley-III = Bayley Scales of Infant and Toddler Development, Third Edition; PDI = motor composite score; FM SS = fine motor scaled score; GM SS = gross motor scaled score.

## Data Availability

Data are available from the corresponding author.
